# Malpractice litigation, workload, and general practitioner retirement

**DOI:** 10.1017/S1463423618000816

**Published:** 2019-03-20

**Authors:** Søren Birkeland, Søren Bie Bogh

**Affiliations:** Centre for Quality and Department of Regional Health Research, University of Southern Denmark, Middelfart, Denmark

**Keywords:** general practice, malpractice, retirement

## Abstract

We investigated the association between general practitioner (GP) stress factors, including involvement in malpractice litigation or high workload levels during 2007 and ensuing retirement in a sample of Danish GPs. The case file and register information of 739 GPs were examined. Hazard ratios (HRs) were estimated for all causes of retirement from 2007 to 2016. During the study period, 34% of GPs had ceased to practice (*n* = 260). The HR for retirement was higher with increasing age (HR = 1.19 per year) and lower if practicing in a clinic with a greater number of GPs (HR = 0.47) but no statistically significant association was found between retirement and litigation or higher workload. Knowledge on factors influencing GPs’ decision on whether to continue working is important to ensure sustainable primary care provision.

## Introduction

In most healthcare systems, patients are entitled to a means of complaining about the quality of the healthcare they receive. While malpractice litigation can be considered a fundamental patient right, it also is challenging for complainants and the healthcare workers who receive the complaint. Medical doctors are concerned about being involved in such cases, and particularly in primary care, the continuous alliance between a general practitioner (GP) and patient is in jeopardy (Birkeland *et al*., 2013). Likewise, research has suggested that both sued and non-sued medical doctors change their professional behavior and emotional reactions in a climate of threat of malpractice litigation, and sued medical doctors show a significantly greater change in these areas than non-sued medical doctors (Charles *et al*., 1985; Mabeck, 2001). Correspondingly, research has found that fear of litigation and actualized malpractice litigation in some doctors may be important factors when considering retirement (Charles *et al*., 1985; Rohrich *et al*., 2008). However, other factors may influence the motivations for GP retirement. For example, manageable workload has been found to be among the incentives for GPs to continue working and likewise GPs in single-handed practices in urban areas seem inclined to retire later (Heath and Sims, 1984; Brett *et al*., 2009).

Research has demonstrated that higher GP seniority is associated with lower patient satisfaction, more malpractice complaints, and with higher risk of being criticized in malpractice complaint cases (Morrison and Wickersham, 1998; Heje *et al*., 2007; Birkeland *et al*., 2013). In addition, research suggests that patient satisfaction is higher for single-handed general practices, and that patient complaints occur more frequently against male GPs and against GPs with higher workloads (Heje *et al*., 2007; Nash *et al*., 2009; Birkeland *et al*., 2013; Birkeland and Bogh, 2017).

As indicated, malpractice litigation in itself is stressful but may pose a particular problem for GPs in single-handed clinics with fewer opportunities to converse with colleagues, and who have extended working hours to serve an oversized patient population. In previous research, time pressure to see patients and the threat of litigation have been found to be among the dominant stressors in GPs’ work lives (Schattner and Coman, 1998). Therefore, a malign spiral may develop with fewer GP resources available for patient care resulting in a greater amount of malpractice litigation. This eventually might be hypothesized to precipitate a GP’s retirement.

We investigated the association between GP retirement statistics and involvement in malpractice litigation, controlling for workload in terms of number of patients listed with the GP, gender, age, location (ie, urban or rural area), and clinic type (single-handed or partnership practice) in a Danish sample of GPs.

## Methods

### Settings

To become a GP in Denmark requires a special educational path. After finishing medical school, a doctor must be authorized as a GP specialist to obtain a contract under the taxation-financed Danish health insurance system. GP specialization takes at least six years, but after specialization, there are no requirements for further education or recertification, and GPs can practice under the contract of the Danish health insurance system until they retire. Almost all Danish citizens are listed with a GP (Birkeland *et al*., 2013). In the Danish healthcare system, GPs act as gatekeepers to the secondary healthcare system. They are responsible for providing healthcare at all hours. Practices are structured either as single-handed practices or partnerships. Currently, there is a development towards having a greater number of partnership practices, which may be explained by an increasing proportion of female GPs who prefer to work in general practices structured as partnerships (Larsen, 2010). Simultaneously, the age of the population of Danish GPs is continuously increasing, and general practice in Denmark is currently confronted with a large proportion of GPs being close to retirement age. Therefore, a deficiency of GPs is developing, particularly in rural areas, which have many single-handed practices (P.L.O., 2017).

In Denmark, patients who are dissatisfied with their GP may initiate malpractice litigation by filing a written complaint to the Danish National Patients Complaints Board (DNPCB). The DNPCB handles complaints about the conduct of healthcare professionals, while compensation claims are handled in a separate organization for Patient Compensation. Similar complaints handling exists in other countries (Bismark *et al*., 2006; Irvine, 2006; Stolper *et al*., 2010). The DNPCB has the authority to impose sanctions in the form of decisions with ‘critiques’, which are mailed to the GP, the complainant and the Danish National Board of Health. Other possible sanctions are ‘critique with injunction’, or bringing the healthcare professional before the prosecuting authority, which can result in withdrawal of the authorization to practice.

### Procedures

Study participants were drawn among all 3765 authorized GPs under contract with the Danish health insurance system in 2007 and for whom information about occupational status was obtainable up to the end of July 2016 (Birkeland *et al*., 2013). This information was found through manual search, predominantly of the Danish state’s master register of information (the ‘Det Centrale Virksomhedsregister’ register), which provides information about businesses. The sample consisted of GPs receiving a complaint *decision* during 2007 and a control group drawn from GPs who had not received a complaint decision from the DNPCB in 2007 (one-10th of all GPs in the Danish health insurance system were randomly selected). We reviewed the DNPCB decisions and information was extracted from case files including the letter of complaint and all documents gathered in the handling process (Birkeland *et al*., 2013). Additionally, we obtained register information about GPs and municipality-level sociodemographic characteristics from the Danish National Board of Health, the Danish Health Information and the Danish Ministry of Welfare databases (Olsen *et al*., 2010). Information on clinic size was dichotomized into single-handed and partnership general practice and location was described on the municipality level as either urban or rural area, according to Organization for Economic Co-operation and Development (OECD) recommendations (threshold set at 150 citizens/km^2^) (Directorate for Public Governance and Territorial Development, 2011; Birkeland and Bogh, 2017).

We used Cox proportional hazard models to test associations between all-cause retirement during the study period and the GP factors discussed above while taking into account clustering effects at the practice level. GPs involved in DNPCB cases were compared to GPs not involved in DNPCB cases. Further analyses were conducted looking at three levels i.e. no litigation, litigation with no critique, and litigation with critique. Time was coded as time-to-event in years from the beginning of 2007. The proportionality–hazard assumption was checked using log–log plots. All analyses were performed using Stata®, release 15.0 (StataCorp, College Station, TX, USA).

## Results

The sample included 739 Danish GPs: 65% (*n* = 477) male and 35% female (*n* = 262). The average age of GPs in January 2007 was 54.3 years. Most practices were partnership clinics (64%, *n* = 472) located in urban areas (52%, *n* = 384). Within the sample, 330 GPs had received a complaint decision during 2007 and 409 GPs had not. In 16% of case decisions (*n* = 53), the GP received a ‘critique’ from the DNPCB. By the end of the study period—end July 2016, 32% had retired (*n* = 234). Statistical analyses of hazard ratios (HRs) for GP retirement during the study period are presented in Table 1.

**Table 1:** Associations between retirement and GP characteristics (*N* = 739)

	HRs for GP retirement between 2007 and July 2016 (95% CI)^a^
Malpractice complaint received	
No	1
Yes	0.88 (0.61–1.27)
Age (per year)	**1.19** (1.15–1.23)
Workload (GP patient list size; per 100 extra patients)	1.05 (0.99–1.11)
Sex	
Female	1
Male	0.91 (0.61–1.34)
Level of urbanization	
Rural (fewer than 150 citizens/km^2^)	1
Urban (at least 150 citizens/km^2^)	0.74 (0.50–1.08)
Type of clinic	
Single-handed	1
Partnership	**0.47** (0.30–0.75)

^a^Boldface values indicate significant results. GP = general practitioner; HR = hazard ratio; CI = confidence interval; *N* = number of observations.

No statistically significant association could be demonstrated between involvement in malpractice litigation in 2007 and GP retirement during the follow-up period. Unsurprisingly, the HR for GP retirement was higher with increasing age of GP (*P* < 0.05). However, practicing in a clinic with a higher number of GPs seemed to be associated with a lower rate of GP retirement (*P* < 0.05). When analyzing GPs subjected to DNPCB critique (as opposed to all GPs involved in DNPCB cases and as opposed to GPs not involved in DNPCB cases), statistically significantly associations again were found with GP age and practice type (*P*<0.05).

## Discussion

In this study, no statistically significant association was found between all-cause retirement and involvement in malpractice complaints and workload but retirement seemingly was postponed for GPs in partnership general practices.

### Discussion of study findings

We initially hypothesized that GPs subjected to malpractice litigation would be likely to retire earlier. Previous research findings suggest this to be true (Charles *et al*., 1985; Rohrich *et al*., 2008). For example, increasing malpractice costs have previously been found to have a strong influence on retirement plans in surgeons 50 years of age and older (Farley *et al*., 2008), and as noted, fear of litigation, actualized malpractice litigation, work overload and lack of staff have been suggested to be factors that may lead to premature GP retirement (Charles *et al*., 1985; Rohrich *et al*., 2008; Brett *et al*., 2009). However, to our knowledge, there exists no previous empirical research on the association between malpractice litigation and realization of retirement plans.

In a study concerning GP registrars’ views on a career in general practice, a great majority of respondents (96%; *N* = 101) expressed an interest in general practice as a career, but increased workload emerged as a negative aspect of general practice. In addition, 99% of the GP registrars agreed that GPs increasingly fear litigation (Rowsell *et al*., 1995).

In this study, there seemed to be a tendency toward GP retiring earlier when having higher workload although no statistically significant association could be established. However, previous findings also suggested that workload influence GPs’ decisions about when to retire (Sansom *et al*., 2016). Our finding that postponed GP retirement is associated with practicing in a partnership structured general practice seems to disagree with our initial hypothesis about the difficulties associated with retiring from a single-handed clinic as well as with former study findings that solo GPs may be inclined to retire later (Heath and Sims, 1984). Experiences from the London area previously revealed a high percentage of single-handed and elderly GPs in clinics with patient lists of more than 2500 patients per GP (McKinnon *et al*., 1999). The reasons for our finding that GPs practicing in a partnership structured general practice retire later are not clear but points to an area that deserves further exploration. It maybe could be hypothesized that professional cooperation and reflection sometimes may postpone retirement plans or prevent ‘burnout’ which sometimes plays a role when GPs decide to retire (Gregory and Menser, 2015). For example, burnout may be accelerated in clinics that have too few GP resources available to serve the patient base (An *et al*., 2009; Chen *et al*., 2013; Oskrochi *et al*., 2016; Nanda *et al*., 2017; Picquendar *et al*., 2018). A previous Danish study among 216 GPs found the seven-year incidence of burnout was 13% and, among 48 GP trainees in the UK, Sales *et al*. demonstrated that signs of burnout develop from the beginning of a GP’s career (Pedersen *et al*., 2013; Sales *et al*., 2016). While patients may prefer the service, accessibility, and facilities of single-handed practices, GPs in single-handed practices may experience higher levels of job stress (McKinnon *et al*., 1999; McDonald *et al*., 2009; van den Hombergh *et al*., 2009).

### Limitations

The variable of GP retirement was measured by a GP retiring from their clinic; however, we found that after leaving their clinic, GPs occasionally served as substitutes in other clinics or had moved into practicing in the hospital sector. It should be noted that only malpractice cases completed during the year 2007 were included in the analysis, and GPs very well may have been involved in malpractice cases before or after this year. Finally, it should be noted that there are several other aspects of GP work life that we have not included in our study (like job satisfaction and variations in work flexibility and patient demands) which may influence GPs’ retirement decisions (Heath and Sims, 1984; Brett *et al*., 2009; Pit and Hansen, 2014).

## Conclusion

Knowledge on the effect of stress factors such as malpractice litigation and work pressure on healthcare workers professional life is important for ensuring sustainable healthcare provision. In this study, we could not document any effect of involvement in malpractice litigation on GP retirement statistics. Instead, our findings suggested that working in single-handed practice influence GP retirement. However, the effect of malpractice litigation and other job-related stressors on medical doctors’ retirement in general practice and in other specialties merits further research.

## Funding

None.

## Conflict of interest

None.
